# The association between predicted inflammatory status and colorectal adenoma

**DOI:** 10.1038/s41598-020-59271-1

**Published:** 2020-02-12

**Authors:** Sejin Kim, Sihan Song, Young Sun Kim, Sun Young Yang, Jung Eun Lee

**Affiliations:** 1 Department of Food and Nutrition, College of Human Ecology, Seoul National University, Seoul, Republic of Korea; 2 Department of Internal Medicine, Healthcare System Gangnam Center, Seoul National University Hospital, Seoul, Republic of Korea; 3 Research Institute of Human Ecology, Seoul National University, 1 Gwanak-ro, Gwanak-gu Seoul, 08826 Republic of Korea

**Keywords:** Cancer epidemiology, Risk factors

## Abstract

We developed a diet and lifestyle score based on high sensitivity C-reactive protein (hsCRP), and investigated its association with odds of adenoma. We performed stepwise linear regression to develop the predicted hsCRP score among 23,330 participants in the Health Examinee Study and examined its association with colorectal adenoma among 1,711 participants in a cross-sectional study of colorectal adenoma. We estimated odds ratios (ORs) and 95% confidence intervals (CIs) of colorectal adenoma using logistic regression models. Variances in hsCRP explained by body mass index were 61.1% in men and 64.5% in women in the prediction model. The increasing predicted hsCRP score was positively associated with colorectal adenoma (OR_quartile 4 VS quartile 1_ 1.71, 95% CI: 1.12–2.62; *P*_trend_ = 0.011 in men; OR_quartile 4 VS quartile 1_ 2.86, 95% CI: 1.26–6.49; *P*_trend_ = 0.019 in women). In subgroups, the associations differed by age and menopausal status among women, with stronger associations among women aged less than 50 years (OR_≥median VS <median_ 3.74, 95% CI: 1.77–7.90, p for interaction 0.014) or premenopausal women (OR_≥median vs <median_ 4.21, 95% CI: 2.12–8.36, p for interaction <0.001). The associations were more pronounced in the advanced or distal colon/rectum in men and in the advanced or proximal colon in women. The associations were attenuated after further adjustment for body mass index. In conclusion, we found that the predicted hsCRP score was positively associated with colorectal adenoma, suggesting that diet and lifestyle lowering inflammation may be a strategy to prevent colorectal neoplasia.

## Introduction

Colorectal cancer has been the third most common cancer in men and the second in women worldwide^1^. In Korea, colorectal cancer was the second most common cancer in men and the third in women^2^. The World Cancer Research Fund (WCRF) reported that being physically active, consuming intakes of whole grains, foods containing dietary fiber and dairy products, and taking calcium supplements decreased the risk of colorectal cancer, while consuming red meat, processed meat and alcohol, and being overweight or obese and tall increased the risk^3^.

Chronic inflammation may play an important role in colorectal neoplasia, considering that chronic inflammation is thought to predispose individuals to cancer^4^. For example, chronic inflammatory conditions, including Crohn’s disease and chronic ulcerative colitis, were risk factors of colorectal carcinoma^5^, whereas nonsteroidal anti-inflammatory drug use reduced the risk of colorectal cancer^6^. Chronic inflammation has been hypothesized to stimulate tumor growth and progression by producing proinflammatory cytokines that activate the transcription factors of tumor cells^4^. Several studies reported that high circulating levels of C-reactive protein (CRP) were associated with risk of colorectal cancer^7^ and higher prevalence of colorectal adenoma^8^, a precancerous lesion of colorectal cancer.

Several studies reported that diet, age, body mass index (BMI), socioeconomic status, and physical activity were linked to inflammatory status^9–16^. Dietary factors in relation to inflammation have been identified in a number of studies exploring a priori or a posteriori dietary patterns^9–12^. Obesity was associated with elevated levels of CRP^13^ as adipocytes synthesize and secrete interleukin-6 (IL-6) and CRP^14^, whereas physical activity lowered levers of CRP^15^. Also, CRP levels differed by age, race, and gender^16^.

A Dietary Inflammatory Index^TM^ (DII^®^) has been recently developed based on the literature review of pro- or anti-inflammatory foods and nutrients^12^, and high scores of DII were positively associated with colorectal cancer risk^17^. Also, an empirically derived dietary pattern that reflected pro-inflammatory status was associated with the risk of colorectal cancer^18^.

In the current study, we developed an index that predicted levels of high-sensitivity C reactive protein (hsCRP), an indicator of chronic inflammation, from foods, nutrients, and lifestyle-related factors in more than 20,000 Korean adults. We further validated the predicted hsCRP score in an independent population, the colorectal adenoma study, and examined whether the predicted hsCRP scores were associated with colorectal adenoma in Korean men and women.

## Material and Methods

### Development of the predicted hsCRP score

#### Study population

We developed the predicted hsCRP score in participants of the Health Examinees (HEXA) Study in Korea, a large-scale genomic population-based study. The HEXA Study forms the largest subcohort of the Korean Genome and Epidemiology Study (KoGES), the principal purpose of which is to investigate epidemiologic characteristics and genomic risk factors for chronic diseases in the Korean population^19^. Participants in the HEXA study were recruited at health examination centers and training hospitals in Korea. A total of 173,357 participants aged 40–79 years were enrolled in the HEXA Study from 2004 to 2013. Details of enrollment and data collection are described elsewhere^20^. In this study, out of the 61,398 participants whose levels of hsCRP were measured with the same analyzer between January, 2004 and October, 2007 and we excluded participants whose hsCRP values were missing (n = 82), and whose hsCRP values were more than 10 mg/L, which is considered acute inflammatory status (n = 1,065)^21^. And we further excluded participants who reported taking hypertension medicine or were diagnosed with hypertension, diabetes, hyperlipidemia, stroke, ischemia, myocardial infarction, or cancer at enrollment (n = 18,829). KoGES provided food frequency questionnaires (FFQs) data after excluding individuals; 1) who did not respond to any questions of FFQs, 2) who left more than 12 blanks for frequency questions, 3) who did not answer any questions about rice intake, or 4) who had extremely low (≤100 kcal/day) or high (≥10,000 kcal/day) energy intake, resulting in exclusion of 1,885 participants. And we further excluded participants who had implausible energy intake ( < 800 or > 4,200 kcal per day for men, <500 or >3,500 kcal per day for women, n = 1,257). Because the disproportionality of sex in the dataset could influence the derivation of the predicted hsCRP score, we included the equal number of men and women by matching men and women by the exact age. As a result, a total of 23,330 participants (11,665 men and 11,665 women) from the HEXA Study were included. All participants provided written informed consent forms to participate in the study. The study was reviewed and approved by the Institutional Review Board of Seoul National University. All of the methods were performed in accordance with the relevant guidelines and regulations.

#### Assessment of the hsCRP levels, diet, and other variables

Blood samples were collected after an 8-hour overnight fast. After the sampling and labeling process, blood samples were centrifuged and stored at 4 °C until analysis. Serum hsCRP levels were measured on a Hitachi 7080 automatic analyzer (Hitachi, Japan) using latex immune complex turbidimetrics (Pure Auto S CRP latex, Daiichi, Japan). The intra-assay coefficient of variation (CV) was 1.63%.

Educated and trained interviewers used a standardized questionnaire survey complying with the study protocol to ask participants about sociodemographic characteristics, including educational level, income, and occupation, medical history, medication use, alcohol intake, smoking status, dietary habits, physical activities, and, for women, reproductive factors.

Participants completed the self-administered 106-item FFQs developed for the Korean population. The reliability of the FFQ has been examined by comparing the dietary intakes from the average amounts based on the first and second FFQ and its validity was examined by comparing 3 dietary records every season, 12-day dietary records (DRs) in total. Pearson correlation coefficients between the FFQ and the 12-day DRs adjusted for age, sex and energy intake were 0.64 for carbohydrate and 0.43 for protein and Pearson correlation coefficients between the first and second FFQs were 0.56 for fat and 0.49 for protein^22^. Nine possible frequency responses, ranging from “not at all or less than once a month” to “three times per day” during the previous one year, were available for each food item. The portion size for each item was reported as one of three sizes: one-half of a standard serving size, one serving size, or one and one-half serving size. Average daily intakes of foods and nutrients were calculated by multiplying the frequency of consumption by the reported amount. To take into account food groups that may be related to inflammation, we classified the 106 items on the FFQ into 38 food groups based on similarity of nutritional characteristics or preparation method (Supplementary Table 1).

We created the model that included thiamin, riboflavin, vitamin B-6, niacin, vitamin A, vitamin C, vitamin E, carbohydrate, total fat, monounsaturated fats, polyunsaturated fats, ω-3 fats, ω -6 fats, saturated fat, protein, fiber, iron, folate, caffeine, total cholesterol, flavanol, anthocyanidins, flavones, flavonols and isoflavones, which showed to be associated with inflammatory biomarkers^12^. We calculated intakes of saturated fat, monounsaturated fatty acid, polyunsaturated fatty acid, ω-3 fats, ω -6 fats, caffeine, flavan-3-ol, flavones, flavonols, anthocyanidins, and isoflavones by referring to the databases of the Rural Development Administration (RDA), the Korea National Health and Nutrition Examination Survey (KNHANES) and the United States Department of Agriculture (USDA). Each nutrient was adjusted by energy intake using the residual method^23^.

BMI was calculated by dividing the participant’s weight (kg) by the square of the height (m^2^). Alcohol intake was estimated by summing up the ethanol weight after multiplying amounts and frequencies of specific types of liquors. Physical activities were estimated by multiplying the frequencies per week and times according to workout types. For missing values of alcohol (0.05%) and BMI (1%), we assigned medians. For missing values of physical activity (3.10%), education level (2.47%) and smoking status (0.69%), participants were assigned to reference groups. If a woman’s menopausal status was not reported (0.84%), we assumed that she was postmenopausal if she was 50 years or older.

#### Development of the predicted hsCRP score

The 38 food groups, nutrients, alcohol intakes, BMI, smoking status, physical activities, educational levels and menopausal status of women were initially included to derive the prediction model of hsCRP because these factors were associated with inflammation^11–13,15,24–27^. The study population was randomly divided into two sets: 70% of the population for a training set and 30% for a testing set. We randomly selected study participants in a sex-specific strata using SAS proc surveyselect (seed number = 499812). The training set was used to develop the score. The testing set was then used to evaluate the validity of the predicted hsCRP score by comparing the actual levels of hsCRP. The levels of hsCRP were log-transformed to improve the normality. We included the aforementioned variables as independent variables and log-transformed hsCRP as a dependent variable in a stepwise linear regression model in the training set, with p = 0.05 as the significance level for entry and retention. Also, we developed indices for men and women combined (sex-combined) and separately (men-specific and women-specific) and compared the potential inflammatory determinants by sex^16,28^.

In the testing set, we computed predicted hsCRP scores by multiplying the individual’s response or estimated intake and the beta coefficient from the derived model. We calculated the least-square mean (LS-mean) for quartiles of the predicted hsCRP scores using the generalized linear model. We then calculated relative concentrations and 95% confidence intervals (CIs) as ratios of LS-mean levels of hsCRP among participants in each subsequent quartile of predicted hsCRP score to those among participants in the lowest quartile. We adjusted for sex (men, women), age (continuous, years), alcohol intake (0, 0- < 15, 15- < 30, ≥30 g/d for men, 0, 0- < 5, 5- < 10, ≥10 g/d for women), smoking status (past, current, never for men, never and ever for women), regular physical exercise (none, <3.5 times per week, ≥3.5 times per week), educational level (elementary school or below, middle school, high school, university or above), and, in women only, menopausal status (premenopausal, perimenopausal or postmenopausal). We additionally adjusted for BMI (continuous, kg/m^2^) in a sensitivity analysis.

### Association between the predicted hsCRP score and colorectal adenoma

#### Study population

Participants in the colorectal adenoma study were 1,056 men and 661 women who underwent colonoscopies for regular health check-ups at Seoul National University Hospital Gangnam Center between May and December 2011^29^. We excluded participants who were diagnosed with colorectal cancer (n = 5); who had a medical history of colorectal cancer (n = 2); or whose energy intakes were implausible (<800 or >4,200 kcal per day for men, <500 or >3,500 kcal per day for women, n = 9). As a result, a total of 1,711 participants (1,056 men and 655 women) were included. We defined participants as having “advanced adenoma” if they had adenomas with villous component, with high-grade dysplasia, in sizes of more than 10 mm, or presence of three or more synchronous adenomas. Colorectal adenomas were divided into proximal colon, distal colon or rectum. The reference point between proximal and distal colon was splenic flexure. All participants provided written informed consent forms to participate in the study. The study was reviewed and approved by the Institutional Review Board of Seoul National University Hospital.

#### Assessment of hsCRP levels, diet, and other variables

Participants were asked about sociodemographic characteristics, alcohol consumption, smoking status, educational levels, physical activities, family history of colorectal cancer, and menopausal status for women only. The participants reported time spent doing vigorous and mild exercise and walking. We calculated a metabolic equivalent task score (METs) for each physical activity. To estimate dietary intakes, participants were asked about the amounts and frequencies of consumption of each food item by a dietitian using the same FFQs validated in KoGES^22^. We directly measured height, weight and waist circumference and calculated BMI. Serum hsCRP was assessed using the ARCHITECT ci16200 (Abbott Laboratories, Abbott Park, IL, USA) automated immunoassay. The intra-assay CV was less than 2%. Participants underwent colonoscopy on the same day as the questionnaire surveys, anthropometric measures and blood draw. According to the colonoscopy findings, participants diagnosed with colorectal adenoma were cases and those without any adenoma were non-cases.

### Statistical analysis

We computed the predicted hsCRP scores by multiplying individual’s response or estimated intake and the beta coefficient derived in a sex-specific way from the HEXA Study. We validated the sex-specific prediction model among a subset of non-cases with hsCRP values (n = 659) in the colorectal adenoma study by calculating the relative concentrations of hsCRP levels according to the predicted hsCRP scores. We calculated the LS-means for quartiles of predicted hsCRP scores using the generalized linear model. Then, we calculated relative concentrations and 95% confidence intervals (CIs) as ratios of LS-mean levels of hsCRP among participants in each subsequent quartile of predicted hsCRP score to those among participants in the lowest quartile. To examine the associations of actual hsCRP levels and predicted hsCRP scores with colorectal adenoma, we calculated ORs and 95% CIs using logistic regression models. We categorized study participants into quartiles according to the predicted hsCRP scores and actual hsCRP levels, respectively. The general characteristics from the colorectal adenoma study population were reported as the means with standard deviations among the continuous variables and as percentages among the categorical variables, according to quartiles of the predicted hsCRP score. In the multivariate model, we adjusted for age (continuous, year), alcohol intake (0, 0- < 15, 15- < 30, 30 ≥g/day for men and 0, 0- < 15, 15 ≥g/day for women), smoking status (past, current, never for men and never and ever for women), physical activity (none, <14, ≥14 METs-hours per week), education levels (high school or less, university or above) and, in women only, menopausal status (premenopausal, postmenopausal). We further adjusted for BMI (continuous, kg/m^2^), as obesity might induce inflammation and be an intermediate factor. The median values of each category were assigned and used as a continuous variable to test the linear trends. We tested for potential effect modifiers by including an interaction term of calculated score classified by median values of the predicted hsCRP score and age, waist circumference, and menopausal status. A likelihood ratio test was used to compare nested models that included cross-product terms with the original models that did not include terms. We used polytomous logistic regression to conduct stratified analyses according to the progress and location of the colorectal adenoma. All statistical analyses were conducted using SAS version 9.4 (SAS Institute Inc., Cary, NC, USA); all tests were two-sided, and P < 0.05 was considered statistically significant.

## Results

### Development of predicted hsCRP score

When we developed the predicted hsCRP model, the components of the prediction model based on the foods, nutrients, and lifestyle related variables differed between the sex-combined model and sex-specific models (Table 1). Age, BMI, and smoking status were selected in all three models (sex-combined, men-specific, and women-specific). Older age, higher BMI, and being a past or current smoker were associated with higher levels of hsCRP. Physical activity was included in the sex-combined and men-specific models, but not in the women-specific model and engagement in exercise was inversely associated with hsCRP levels. Education levels and menopausal status remained only in the women-specific model. Regarding dietary factors, higher levels of hsCRP were associated with: higher intakes of alcohol, breakfast cereals/mixed grain powder, noodles/dumplings, potatoes, beef, and carbonated beverages; and, lower intakes of sweet bread, soup and stew with soybean paste/soybean paste, sweet potatoes, and fruits in the sex-combined model. Dietary factors selected in the men-specific model were different from those in the women-specific model. Among men only, there were positive associations for intakes of niacin and noodles/dumplings and inverse associations for intakes of sweet potatoes and soup and stew with soybean paste/soybean paste. In the women-specific model, increasing intakes of beef and processed fish and decreasing intakes of fish, soup and stew with soybean paste/soybean paste and sweet bread were associated with increasing levels of hsCRP. Variances in hsCRP explained by BMI were 61.1% in men and 64.5% in women in the prediction model.

**Table 1:** Components of the predicted hsCRP scores based on foods, nutrients and lifestyle factors in sex-combined and sex-specific model.

Sex-combined			Men-specific			Women-specific		
Variables	Beta	p value	Variables	Beta	p value	Variables	Beta	p value
**Positively associated**								
Alcohol intake^a^ (g/d)	0.0009	0.002	Niacin (mg/d)	0.1360	0.002	Beef (g/d)	0.0009	0.040
Breakfast cereals/mixed grain powder (g/d)	0.0015	0.035	Noodles/dumplings (g/d)	0.0004	<0.001	Processed fish (g/d)	0.0028	0.013
Noodles/dumplings (g/d)	0.0003	<0.001	Age (y)	0.0113	<0.001	Age (y)	0.0140	<0.001
Potatoes (g/d)	0.0012	0.016	BMI (1 kg/m^2^)	0.0707	<0.001	BMI (1 kg/m^2^)	0.0782	<0.001
Beef (g/d)	0.0011	<0.001	Smoking status			Smoking status		
Carbonated beverages (g/d)	0.0003	0.018	Never	Reference		Never	Reference	
Age (y)	0.0158	<0.001	Past smoker	0.0370	0.081	Past smoker	0.1514	0.056
BMI (1 kg/m^2^)	0.0773	<0.001	Current smoker	0.1990	<0.001	Current smoker	0.1360	0.016
Smoking status						Menopausal status		
Never	Reference					Premenopausal	Reference	
Past smoker	0.0787	<0.001				Perimenopausal	0.0587	0.043
Current smoker	0.2547	<0.001				Postmenopausal	0.1576	<0.001
Negatively associated								
Soup and stew with soybean paste/soybean paste (g/d)	−0.0042	<0.001	Soup and stew with soybean paste/soybean paste (g/d)	−0.0055	0.002	Soup and stew with soybean paste/soybean paste (g/d)	−0.0033	0.031
Sweet potatoes (g/d)	−0.0010	0.007	Sweet potatoes (g/d)	−0.0017	0.017	Sweet bread (g/d)	−0.0010	0.020
Sweet bread (g/d)	−0.0007	0.035	Exercise			Fish (g/d)	−0.0007	0.014
Fruits (g/d)	−0.0001	0.020	None	Reference		Educational level		
Exercise			0 < - < 3.5 times/d	−0.1283	<0.001	Elementary school or below	Reference	
None	Reference		≥3.5 times/d	−0.1002	<0.001	Middle school	−0.0659	0.010
0 < - < 3.5 times/d	−0.0586	<0.001				High school	−0.0256	0.271
≥3.5 times/d	−0.0707	<0.001				University or above	0.0247	0.395

^a^Alcohol intake was estimated by summing up the ethanol weight after multiplying amounts and frequencies of specific types of alcoholic beverages.

The food group included the following food items: breakfast cereals/mixed grain powder, breakfast cereals and mixed grain powder; noodles/dumplings, noodles, instant noodles, noodles in blackbean sauce, spicy seafood noodle soup, cold noodles, dumplings, and japchae; soup and stew with soybean paste/soybean paste, soup and stew with soybean paste, soybean paste, and seasoning soybean paste; sweet bread, red bean bread, and doughnuts; fruits, tangerine, orange, strawberries, watermelon, apples, pear, bananas, and grapes; processed fish, canned tuna fish and fish cake.

We found that the relative concentrations of the actual levels of hsCRP in the testing set increased according to increasing quartiles of the predicted hsCRP score (Table 2). In the sex-combined model, the relative concentrations (95% CIs) for the highest compared with the lowest predicted hsCRP score were 1.82 (95% CI: 1.66–2.00) for men and women combined, 1.64 (95% CI: 1.46–1.83) among men and 1.90 (95% CI: 1.65–2.19) among women. When we estimated the relative concentrations using the men-specific and women-specific models, the relative concentrations comparing participants with the highest predicted hsCRP score and the lowest predicted hsCRP score were 1.65 (95% CI: 1.49–1.84) among men and 2.02 (95% CI: 1.74–2.34) among women. When we further adjusted for BMI, the relative concentrations of the highest predicted hsCRP score were 1.17 (95% CI: 0.98–1.40) among men and 1.14 (95% CI: 0.93–1.41) among women in sex-specific models.

**Table 2:** Relative concentrations and 95% confidence intervals between the predicted hsCRP scores and the actual hsCRP levels in the testing set of the HEXA.

	Quartiles of the predicted hsCRP score				*p* for trend
	Quartile 1	Quartile 2	Quartile 3	Quartile 4	
**Sex-combined model (n = 7,108)**					
hsCRP, mg/L, mean ± SD	0.73 ± 1.02	0.99 ± 1.16	1.23 ± 1.39	1.44 ± 1.47	
Age, sex adjusted model	Reference	1.30 (1.21, 1.41)	1.55 (1.44, 1.67)	1.84 (1.70, 1.99)	<0.001
Multivariate adjusted model^a^	Reference	1.30 (1.18, 1.43)	1.54 (1.41, 1.69)	1.82 (1.66, 2.00)	<0.001
Multivariate adjusted model^b^	Reference	1.08 (0.95, 1.23)	1.13 (1.00, 1.27)	1.11 (0.96, 1.27)	0.084
** Men in sex-combined model (n = 3,554)**					
hsCRP, mg/L, mean ± SD	0.94 ± 1.14	1.21 ± 1.42	1.22 ± 1.29	1.50 ± 1.46	
Age-adjusted model	Reference	1.27 (1.15, 1.41)	1.35 (1.21, 1.49)	1.68 (1.51, 1.86)	<0.001
Multivariate adjusted model^c^	Reference	1.26 (1.13, 1.41)	1.32 (1.19, 1.48)	1.64 (1.46, 1.83)	<0.001
Multivariate adjusted model^d^	Reference	1.10 (0.95, 1.27)	1.05 (0.91, 1.21)	1.12 (0.94, 1.34)	0.292
** Women in sex-combined model (n** = **3,554)**					
hsCRP, mg/L, mean ± SD	0.63 ± 0.89	0.86 ± 1.07	1.02 ± 1.16	1.41 ± 1.59	
Age-adjusted model	Reference	1.27 (1.14, 1.42)	1.44 (1.30, 1.60)	1.85 (1.65, 2.07)	<0.001
Multivariate adjusted model^e^	Reference	1.28 (1.12, 1.47)	1.46 (1.27, 1.67)	1.90 (1.65, 2.19)	<0.001
Multivariate adjusted model^f^	Reference	1.07 (0.90, 1.26)	1.05 (0.89, 1.24)	1.08 (0.89, 1.32)	0.405
** Men-specific model (n** = **3,560)**					
hsCRP, mg/L, mean ± SD	0.93 ± 1.18	1.10 ± 1.26	1.15 ± 1.28	1.48 ± 1.39	
Age-adjusted model	Reference	1.21 (1.10, 1.34)	1.28 (1.16, 1.42)	1.69 (1.52, 1.86)	<0.001
Multivariate adjusted model^c^	Reference	1.20 (1.08, 1.34)	1.26 (1.14, 1.41)	1.65 (1.49, 1.84)	<0.001
Multivariate adjusted model^d^	Reference	1.06 (0.92, 1.22)	1.02 (0.89, 1.18)	1.17 (0.98, 1.40)	0.120
** Women-specific model (n** = **3,560)**					
hsCRP, mg/L, mean ± SD	0.63 ± 0.89	0.82 ± 1.01	1.08 ± 1.37	1.41 ± 1.52	
Age-adjusted model	Reference	1.25 (1.12, 1.39)	1.51 (1.35, 1.69)	1.97 (1.75, 2.21)	<0.001
Multivariate adjusted model^e^	Reference	1.27 (1.11, 1.47)	1.55 (1.35, 1.79)	2.02 (1.74, 2.34)	<0.001
Multivariate adjusted model^f^	Reference	1.06 (0.89, 1.26)	1.11 (0.93, 1.32)	1.14 (0.93, 1.41)	0.133

^a^Adjusted for sex (men, women), age (continuous, years), alcohol (0, 0- < 15, 15- < 30, ≥30 g/d), smoking status (past, current, never), regular physical exercise (none, <3.5 times per week, ≥3.5 times per week), educational level (elementary school or below, middle school, high school, university or above).

^b^Adjusted for sex (men, women), age (continuous, years), alcohol (0, 0- < 15, 15- < 30, ≥30 g/d), smoking status (past, current, never), regular physical exercise (none, <3.5 times per week, ≥3.5 times per week), educational level (elementary school or below, middle school, high school, university or above), and BMI (continuous, kg/m^2^).

^c^Adjusted for age (continuous, years), alcohol (0, 0- < 15, 15- < 30, ≥30 g/d), smoking status (past, current, never), regular physical exercise (none, <3.5 times per week, ≥3.5 times per week), educational level (elementary school or below, middle school, high school, university or above).

^d^Adjusted for age (continuous, years), alcohol (0, 0- < 15, 15- < 30, ≥30 g/d), smoking status (past, current, never), regular physical exercise (none, <3.5 times per week, ≥3.5 times per week), educational level (elementary school or below, middle school, high school, university or above), and BMI (continuous, kg/m^2^).

^e^Adjusted for age (continuous, years), alcohol (0, 0 < - < 5, 5- < 10, ≥10 g/d), smoking status (ever, never), regular physical exercise (none, <3.5 times per week, ≥3.5 times per week), educational level (elementary school or below, middle school, high school, university or above), and menopausal status (postmenopausal, perimenopausal, postmenopausal).

^f^Adjusted for age (continuous, years), alcohol (0, 0 < - < 5, 5- < 10, ≥10 g/d), smoking status (ever, never), regular physical exercise (none, <3.5 times per week, ≥3.5 times per week), educational level (elementary school or below, middle school, high school, university or above), menopausal status (postmenopausal, perimenopausal, postmenopausal), and BMI (continuous, kg/m^2^).

### Association between predicted hsCRP score and colorectal adenoma

The general characteristics of men and women by quartiles of the predicted hsCRP scores are presented in Table 3. Men who had the higher predicted hsCRP score were more likely to be older, current smokers and to have higher BMI. Men in the 3^rd^ or 4^th^ quartiles had lower proportions of university or above education and 14 or greater METs-hours per week of exercise compared to those in the 1^st^ or 2^nd^ quartiles. Women who had the higher predicted hsCRP scores tended to be older, postmenopausal and to have higher BMI and lower proportions of university or above education compared to those with lower scores.

**Table 3:** Characteristics by quartiles of the predicted hsCRP score using sex-specific models among men and women in the colorectal adenoma study.

	Quartiles of the predicted hsCRP score			
	Quartile 1	Quartile 2	Quartile 3	Quartile 4
**Men (n = 1,056)**	(n = 264)	(n = 264)	(n = 264)	(n = 264)
Number of cases/non-cases	75/189	98/166	110/154	123/141
Age (years, %)	47.9 ± 8.0	51.4 ± 8.4	52.6 ± 8.1	54.5 ± 9.4
<50 years	141 (53.4)	107 (40.5)	98 (37.1)	73 (27.7)
≥50 years	123 (46.6)	157 (59.5)	166 (62.9)	191 (72.4)
Smoking stats (%)				
Never	103 (39.5)	67 (25.8)	57 (22.1)	35 (13.4)
Past smoker	123 (47.1)	137 (52.7)	107 (41.5)	97 (37.2)
Current smoker	35 (13.4)	56 (21.5)	94 (36.4)	129 (49.4)
BMI (kg/m^2^)	21.9 ± 1.8	23.8 ± 1.4	25.1 ± 1.6	27.2 ± 2.0
Educational level (%)				
High school or less	22 (8.0)	37 (14.5)	40 (16.1)	46 (18.2)
University or above	233 (91.4)	219 (85.5)	209 (83.9)	207 (81.8)
Alcohol intake (%)				
0 g	22 (8.5)	25 (9.7)	32 (12.7)	30 (11.8)
0 g < - < 15 g	109 (42.1)	87 (33.7)	66 (26.1)	74 (29.0)
15 g ≤ - < 30 g	55 (21.2)	59 (22.9)	64 (25.3)	50 (19.6)
30 g≤	73 (28.2)	87 (33.7)	91 (36.0)	101 (39.6)
Exercise (%)				
None	62 (23.9)	87 (33.5)	114 (44.7)	127 (49.0)
0- < 14 METs-hours/week	81 (31.2)	58 (22.3)	44 (17.3)	29 (11.2)
≥14 METs-hours/week	117 (45.0)	115 (44.2)	97 (38.0)	103 (39.8)
**Women (n = 655)**	(n = 163)	(n = 164)	(n = 164)	(n = 164)
Number of cases/non-cases	14/149	31/133	48/116	56/108
Age (years, %)	41.8 ± 5.4	47.8 ± 6.0	53.4 ± 6.7	58.1 ± 7.8
<50 years	146 (89.6)	100 (61.0)	40 (24.4)	19 (12.0)
≥50 years	17 (10.4)	64 (39.0)	124 (75.6)	145 (88.4)
Smoking status (%)				
Never	149 (92.6)	145 (90.1)	154 (95.7)	142 (87.7)
Past smoker	5 (3.1)	11 (6.8)	4 (2.5)	11 (6.8)
Current smoker	7 (4.4)	5 (3.1)	3 (1.9)	9 (5.6)
BMI (kg/m^2^)	19.4 ± 1.3	21.2 ± 1.6	22.3 ± 1.8	25.3 ± 3.1
Post-menopausal status (%)	8 (5.1)	51 (31.9)	108 (67.5)	136 (84.0)
Educational level (%)				
High school or less	26 (16.7)	37 (24.2)	48 (31.6)	61 (39.9)
University or above	130 (83.3)	116 (75.8)	104 (68.4)	92 (60.1)
Alcohol intake (%)				
0 g	66 (42.3)	67 (42.4)	73 (46.5)	93 (60.0)
0 g < - < 15 g	77 (49.4)	70 (44.3)	71 (45.2)	47 (30.3)
15 g ≤ - < 30 g	7 (4.5)	9 (5.7)	8 (5.1)	10 (6.5)
30 g≤	6 (3.9)	12 (7.6)	5 (3.2)	5 (3.2)
Exercise (%)				
None	74 (46.5)	73 (45.9)	79 (50.0)	80 (50.0)
0- < 14 METs-hours/week	36 (22.6)	26 (16.4)	30 (19.0)	30 (18.8)
≥14 METs-hours/week	49 (30.8)	60 (37.7)	49 (31.0)	50 (31.3)

Data are expressed as arithmetic mean ± SD if not stated otherwise.

When we estimated the relative concentrations of actual hsCRP levels in the colorectal adenoma study, the relative concentrations comparing participants with the highest predicted hsCRP score and the lowest predicted hsCRP score were 2.13 (95% CI: 1.43–3.17; *P* for trend < 0.001) among men and 2.82 (95% CI: 1.58–5.03; *P* for trend < 0.001) among women (Table 4). When we additionally adjust for BMI, trend became non-significant.

**Table 4:** Relative concentrations and 95% CIs between the predicted hsCRP scores of sex-specific models and actual hsCRP levels among non-case participants in the colorectal adenoma study.

	Quartiles of the predicted hsCRP score				*p* for trend
	Quartile 1	Quartile 2	Quartile 3	Quartile 4	
**Men (n = 352)**					
hsCRP, mg/L, mean ± SD	0.24 ± 0.07	0.49 ± 0.08	0.90 ± 0.17	2.71 ± 1.67	
Age-adjusted model	Reference	1.42 (1.01, 2.01)	1.61 (1.12, 2.32)	1.96 (1.35, 2.86)	< 0.001
Multivariate adjusted model^a^	Reference	1.46 (1.01, 2.09)	1.77 (1.20, 2.59)	2.13 (1.43, 3.17)	< 0.001
Multivariate adjusted model^b^	Reference	1.04 (0.65, 1.66)	1.05 (0.63, 1.75)	0.94 (0.50, 1.79)	0.859
**Women (n = 293)**					
hsCRP, mg/L, mean ± SD	0.17 ± 0.05	0.38 ± 0.08	0.81 ± 0.18	2.54 ± 1.68	
Age-adjusted model	Reference	1.47 (0.92, 2.34)	1.56 (0.98, 2.48)	2.45 (1.45, 4.13)	< 0.001
Multivariate adjusted model^c^	Reference	1.54 (0.92, 2.59)	1.71 (1.02, 2.85)	2.82 (1.58, 5.03)	< 0.001
Multivariate adjusted model^d^	Reference	1.19 (0.62, 2.29)	1.11 (0.57, 2.15)	1.38 (0.57, 3.33)	0.554

^a^Adjusted for age (continuous, years), alcohol (0, 0- < 15, 15- < 30, ≥30 g/d), smoking status (past, current, never), regular physical exercise (none, <14 METs-hours/week, ≥14 METs-hours/week), and educational level (high school or below, university or above).

^b^Adjusted for age (continuous, years), alcohol (0, 0- < 15, 15- < 30, ≥30 g/d), smoking status (past, current, never), regular physical exercise (none, <14 METs-hours/week, ≥14 METs-hours/week), educational level (high school or below, university or above), and BMI (continuous, kg/m^2^).

^c^Adjusted for age (continuous, years), alcohol (0, 0- < 15, ≥15 g/d), smoking status (ever, never), regular physical exercise (none, <14 METs-hours/week, ≥14 METs-hours/week), educational level (high school or below, university or above), and menopausal status (premenopausal, postmenopausal).

^d^Adjusted for age (continuous, years), alcohol (0, 0- < 15, ≥15 g/d), smoking status (ever, never), regular physical exercise (none, <14 METs-hours/week, ≥14 METs-hours/week), educational level (high school or below, university or above), menopausal status (premenopausal, postmenopausal), and BMI (continuous, kg/m^2^).

When we examined the association between actual hsCRP levels and colorectal adenoma, we found that increasing levels of actual hsCRP were associated with increasing prevalence of colorectal adenoma in men (*P* for trend = 0.020) and women (*P* for trend = 0.003)(Supplementary Table 2).

We found that increasing predicted hsCRP scores were associated with increasing prevalence of colorectal adenoma (Table 5). Compared with participants in the lowest quartile, the ORs of colorectal adenoma among those in the highest quartile of the predicted hsCRP score were 1.71 (95% CI: 1.12–2.62; *P* for trend = 0.011) among men and 2.86 (95% CI: 1.26–6.49; *P* for trend = 0.019) among women. When we further adjusted for BMI, the ORs comparing the highest quartiles with the lowest quartiles of the predicted hsCRP score were attenuated to 0.98 (95% CI: 0.42–2.31) in men and 1.61 (95% CI: 0.46–5.64) in women.

**Table 5:** Odds ratio (ORs) and 95% confidence interval (CIs) for colorectal adenoma according to quartiles of the predicted hsCRP score of men-specific and women-specific models.

	Quartiles of the predicted hsCRP score				*p* for trend
	Quartile 1	Quartile 2	Quartile 3	Quartile 4	
**Men (n = 1,056)**					
Number of case/noncase	75/189	98/166	110/154	123/141	
Age-adjusted model	Reference	1.27 (0.87, 1.85)	1.46 (1.00, 2.12)	1.63 (1.12, 2.38)	0.009
Multivariate adjusted model^a^	Reference	1.30 (0.89, 1.91)	1.52 (1.02, 2.27)	1.71 (1.12, 2.62)	0.011
Multivariate adjusted model^b^	Reference	1.06 (0.66, 1.70)	1.08 (0.59, 1.98)	0.98 (0.42, 2.31)	0.974
**Women (n = 655)**					
Number of case/noncase	24/139	30/134	37/127	58/106	
Age-adjusted model	Reference	2.03 (1.01, 4.06)	2.97 (1.44, 6.10)	3.15 (1.44, 6.91)	0.007
Multivariate adjusted model^c^	Reference	1.88 (0.93, 3.81)	2.87 (1.36, 6.03)	2.86 (1.26, 6.49)	0.019
Multivariate adjusted model^d^	Reference	1.57 (0.73, 3.37)	2.07 (0.83, 5.16)	1.61 (0.46, 5.64)	0.512

^a^Adjusted for age (continuous, years), alcohol (0, 0- < 15, 15- < 30, ≥30 g/d), smoking status (past, current, never), regular physical exercise (none, <14 METs-hours/week, ≥14 METs-hours/week), and educational level (high school or below, university or above).

^b^Adjusted for age (continuous, years), alcohol (0, 0- < 15, 15- < 30, ≥30 g/d), smoking status (past, current, never), regular physical exercise (none, <14 METs-hours/week, ≥14 METs-hours/week), educational level (high school or below, university or above), and BMI (continuous, kg/m^2^).

^c^Adjusted for age (continuous, years), alcohol (0, 0- < 15, ≥15 g/d), smoking status (past/current, never), regular physical exercise (none, <14 METs-hours/week, ≥14 METs-hours/week), educational level (high school or below, university or above), and menopausal status (premenopausal, postmenopausal).

^d^Adjusted for age (continuous, years), alcohol (0, 0- < 15, ≥15 g/d), smoking status (past/current, never), regular physical exercise (none, <14 METs-hours/week, ≥14 METs-hours/week), educational level (high school or below, university or above), menopausal status (premenopausal, postmenopausal), and BMI (continuous, kg/m^2^).

We examined whether the associations between the predicted hsCRP scores and colorectal adenoma were modified by age, waist circumference and menopausal status (Table 6). Significant differences were not observed when stratified by waist circumference in either men or women. The interactions by age and menopausal status were significant among women. When we stratified women by age (<50 or ≥50 years), the ORs (95% CIs) comparing equal to and more than median values of predicted hsCRP score with under the median values were 3.74 (95% CI: 1.77–7.90) for women who were under 50 years and 1.09 (95% CI: 0.57–2.07) for women who were 50 years or older (p for interaction = 0.014). The ORs for comparing equal to and more than median values of predicted hsCRP score with under the median values were 4.21 (95% CI: 2.12–8.36) for premenopausal women and 0.71 (95% CI: 0.36–1.41) for postmenopausal women (p for interaction <0.001).

**Table 6:** Odds ratio (OR)s and 95% confidence interval (CI)s according to the predicted hsCRP, stratified by risk factors.

	Dichotomous category of the predicted hsCRP scores				*P* for interaction
	<median		≥median		
	No. cases/non-cases	OR (95% CI)	No. cases/non-cases	OR (95% CI)	
**Men**^a^	173/355		233/295		
Age					
<52 years, median	79/221	Reference	72/137	1.41 (0.91, 2.20)	0.801
≥52 years	94/134	Reference	161/158	1.42 (0.97, 2.10)	
Waist circumference					
<90 cm	151/309	Reference	77/110	1.10 (0.72, 1.70)	0.208
≥90 cm	21/41	Reference	143/183	1.19 (0.63, 2.26)	
**Women**^b^	45/282		104/224		
Age					
<50 years, median	26/220	Reference	19/40	3.74 (1.77, 7.90)	0.014
≥50 years	19/62	Reference	85/184	1.09 (0.57, 2.07)	
Waist circumference					
<80 cm	34/212	Reference	25/57	0.89 (0.39, 2.02)	0.651
≥80 cm	10/67	Reference	77/158	3.17 (1.40, 7.18)	
Menopausal status					
pre-menopause	26/232	Reference	26/52	4.21 (2.12, 8.36)	< 0.001
post-menopause	18/41	Reference	74/170	0.71 (0.36, 1.41)	

^a^Adjusted for age (continuous, years), alcohol (0, 0- < 15, 15- < 30, ≥30 g/d), smoking status (past, current, never), regular physical exercise (none, <14 METs-hours/week, ≥14 METs-hours/week), and educational level (high school or below, university or above).

^b^Adjusted for age (continuous, years), alcohol (0, 0- < 15, ≥15 g/d), smoking status (past, current, never), regular physical exercise (none, <14 METs-hours/week, ≥14 METs-hours/week), educational level (high school or below, university or above), and menopausal status (premenopausal, postmenopausal).

We further examined the association between the predicted hsCRP score and colorectal adenoma according to progressive stage and location (Table 7). Stronger associations between the predicted hsCRP scores and advanced adenoma were observed in both men (OR: 1.62, 95% CI: 1.00–2.63) and women (OR: 6.55, 95% CI: 1.62–26.37). When we additionally adjusted for BMI, ORs (95% CIs) were 1.30 (95% CI: 0.67–2.52) among men and 3.51 (95% CI: 0.75–16.40) among women. When stratified by anatomical sites among men, the association was statistically significant for distal colon and rectal adenomas (OR: 1.83, 95% CI: 1.21–2.77), but not for proximal colon adenomas. Whereas among women, the association was stronger for proximal colon adenoma than for distal colon and rectal adenomas. Women with median or higher values of the predicted hsCRP scores had a 1.95 times higher prevalence of proximal colon adenoma compared to those with lower than median values.

**Table 7:** Odds ratio (OR)s and 95% confidence interval (CI)s according to the predicted hsCRP score, stratified by progression and location.

	Dichotomous category of the predicted hsCRP score			
	Men (n = 1,056)^a^		Women (n = 655)^b^	
	No. cases/non-cases	OR (95% CI)	No. cases/non-cases	OR (95% CI)
**All colorectal adenoma**				
<median	173/355	Reference	45/282	Reference
≥median	233/295	1.44 (1.10, 1.89)	104/224	1.86 (1.13, 3.06)
**Non-advanced adenoma**				
<median	137/355	Reference	42/282	Reference
≥median	155/295	1.30 (0.95, 1.79)	80/224	1.49 (0.87, 2.55)
**Advanced adenoma**				
<median	36/355	Reference	3/282	Reference
≥median	78/295	1.62 (1.00, 2.63)	24/224	6.55 (1.62, 26.37)
**Proximal colon**				
<median	121/355	Reference	23/282	Reference
≥median	131/295	1.16 (0.83, 1.62)	62/224	1.95 (1.02, 3.75)
**Distal colon and rectum**				
<median	52/355	Reference	22/282	Reference
≥median	102/295	1.83 (1.21, 2.77)	42/224	1.65 (0.82, 3.33)

^a^Adjusted for age (continuous, years), alcohol (0, 0- < 15, 15- < 30, ≥30 g/d), smoking status (past, current, never), regular physical exercise (none, <14 METs-hours/week, ≥14 METs-hours/week), and educational level (high school or below, university or above).

^b^Adjusted for age (continuous, years), alcohol (0, 0- < 15, ≥15 g/d), smoking status (past, current, never), regular physical exercise (none, <14 METs-hours/week, ≥14 METs-hours/week), educational level (high school or below, university or above), and menopausal status (premenopausal, postmenopausal).

## Discussion

In this cross-sectional study, we derived the predicted score to reflect chronic inflammatory status. We found that the predicted hsCRP scores were correlated with actual hsCRP levels in the colorectal adenoma study participants, suggesting that the predicted hsCRP scores may reflect inflammatory status in Korean adult populations. We found that men and women with high predicted hsCRP scores had higher prevalence of colorectal adenoma compared to those with low scores. The associations were more pronounced among women aged less than 50 years or premenopausal. Men and women with high predicted hsCRP scores had higher prevalence of advanced colorectal adenoma compared to those with low predicted scores, but this association was not observed for non-advanced adenoma.

We found that the higher intakes of noodles/dumplings, beef, breakfast cereals/mixed grain powder, potatoes, carbonated beverages, and processed fish and the lower intakes of soybean paste/soup and stew with soybean paste, sweet potatoes, sweet breads, fruits, and fish were associated with increased levels of hsCRP. Our findings for dietary factors related to inflammation corroborate the results of other previous studies. In the empirically derived inflammatory pattern of the Nurses’ Health Study, higher intakes of processed meat, red meat, organ meat, refined grains and high-energy beverages and lower intakes of dark yellow vegetables including sweet potatoes, snacks, and fruit juice were associated with increasing levels of CRP, IL-6, and tumor necrosis factor-alpha(TNF-α)^11^. Several studies reported that higher intakes of red meat^30–33^ and soft drinks^30,32,34–36^ and lower intakes of fruits^32,36^, soy foods/legumes^34,37^, and dark and yellow vegetables^30,32,34–36^ were associated with increasing levels of inflammatory markers such as CRP, IL-6, and TNF-α. Also, a Korean case-control study found an association between inflammatory dietary pattern and risk of colorectal cancer^38^. In that study, high scores of the CRP-dietary pattern scores were positively associated with the intakes of grains, salted fermented seafood, carbonated beverages, seafood/seashell, oils, noodles, and sweets. In contrast, the intakes of fruits, bonefish, vegetables, milk, nuts, tubers, tea/beverages, seaweeds, and condiments/seasonings were inversely associated with the dietary pattern scores.

When we compared the sex-combined and sex-specific models, we observed that the components of the prediction models and the magnitude of the relative concentrations differed by sex. Although differences of CRP by sex were controversial, it was reported that levels of hsCRP in women were higher than men in the U.S. population^16,39^. In contrast, men had higher CRP levels than both pre- and postmenopausal women in Japanese^40^ and Korean population^28^. It is well-known that men and women have different physical and physiological characteristics, for example, body composition and sex hormones^41^. *In vivo* and *in vitro* studies found that endogenous sex steroids might act as inflammatory regulators in the inflammatory processes^42.^ Sex differences in components related to hsCRP levels may be partly explained by biological difference. Also, sex difference that we found could be due to differences in dietary intakes^43,44^. A previous Korean study found sex differences in the amount of food and selection of food items in the KNHNAES^43^.

We observed that higher values of the actual hsCRP and predicted hsCRP scores were associated with higher prevalence of the colorectal adenoma in both men and women. However, further adjustment for BMI attenuated the associations between hsCRP levels and colorectal adenoma. The reason why we found the attenuation after further adjustment for BMI might be because BMI was a strong determinant for hsCRP levels.

Chronic inflammation contributes to development and progression of cancer^4^. Chronic inflammation activates the transcription factors such as NF-κB and signal transducer and activator of transcription 3 (STATA3) of tumor cells^45^. These activated transcription factors stimulate production of cytokines and chemokines, resulting in recruitment of various leukocytes^4^. This leads to cell proliferation, angiogenesis and lymphangiogenesis and invasion of tumor cells^46^. A recent meta-analysis has revealed that elevated CRP levels were associated with colorectal cancer^7^ and colorectal advanced adenoma^8^. The DII^TM^ was developed based on the literature review^12^ and was found to be positively associated with prevalence of colorectal adenoma^47^ and the risk of colorectal cancer^17^. The Nurses’ Health Study reported that the hazard ratios (HRs) of the highest quintile of empirical dietary inflammatory pattern scores compared to the lowest were 1.44(95% CI: 1.19–1.74; *P* for trend < 0.001) among men and 1.22 (95% CI: 1.02–1.45; *P* for trend = 0.007) among women^18^.

In the prediction models, BMI, age, and smoking status were selected as determinants for hsCRP levels in both men and women. Obesity is associated with chronic inflammation^13^. Adipocytes produce inflammation-related factors such as IL-6, TNF-α, and adiponectin^48^. The overexpression of pro-inflammatory cytokines and IL-6 stimulates hepatocytes and drives the systemic inflammation in the body^49^. Oxidative stress produced from the cigarette burning and the aging process induces chronic upregulation of pro-inflammatory mediators activating the NF-κB signaling pathway^50,51^. These inflammatory mediators recruit chronic immune cells and promote inflammation^50,51^.

In our study, physical activity in men-specific models and education level and menopausal status in women-specific models were included. Physical activity was significantly inversely associated with CRP in British men^52^. Regular exercise reduced toll-like receptor 4 (TLR4) expression and lowered lipopolysaccharide-stimulated IL-6 production^53^. Additionally, participants whose educational levels were college or above had lower CRP levels compared to those whose educational levels were high school or below^26^. The Women’s Health Study has reported inflammatory markers increased from being premenopausal to postmenopausal. The increase in visceral adiposity across the menopausal transition contributes to increasing the inflammation levels^54^.

We found that the predicted hsCRP scores were positively associated with colorectal adenoma among women who were premenopausal and under 50 years old. Our findings are consistent with previous studies that examined the association between BMI and colorectal status by age and menopausal status^55–58^. Those studies found positive associations only among young women^55,56^ or among premenopausal women^57^. A Chinese case-control study reported that increasing prevalence of colorectal cancer was associated with increasing BMI among premenopausal women, while decreasing prevalence of colorectal cancer was associated with increasing BMI among postmenopausal women. Previous findings suggested that menopausal status could be an important effect modifier for colorectal cancer development^58^.

More pronounced association for advanced adenoma observed in our study is consistent with findings from previous studies. The Tennessee Colorectal Polyp Study in the U.S. observed the stronger association between CRP levels and multiple small tubular or advanced adenomas^59^. Participants in the highest tertile had a 2.01 times higher prevalence of advanced adenoma compared to those in the lowest tertile. Two other studies in Japanese also found that the circulating levels of CRP were positively associated with the prevalence odds of advanced or large (≥5 mm) adenomas^60,61^.

Whether the association with either circulating CRP levels or inflammatory scores varied by adenoma sites was not consistent^60,62,63^. Inverse association between CRP levels and proximal colon, but positive association for distal colon adenoma in the CLUE II cohort study^62^. A Japanese case-control study found that the associations for CRP levels were not different by sites of colorectal adenomas^60^. The Nurses’ Health Study showed that increasing CRP levels were only associated with increasing proximal colon, not with distal colon and rectum^63^. The Women’s Health Initiative Study reported that increase in colon cancer risk with increasing levels of DII was limited to proximal colon^64^. In the US male cohort study, men with high predicted CRP scores derived by reduced rank regression had a higher risk of colon, proximal, distal and rectal cancers^18^. The Nurses’ Health Study also found that increasing predicted CRP scores were associated with increasing risk of colon, proximal, and distal cancers^18^. Likewise, inconsistent findings were observed in other studies^65–68^.

Our study had several strengths. The inflammatory prediction model was derived from more than 20,000 healthy participants. We validated the predicted hsCRP score both in the testing set and in the independent population with actual hsCRP levels. This study included more than 1,700 Korean participants who underwent colonoscopies, which enabled us to examine the entire colon. Our study also had some limitations. First, because this was a cross-sectional study, our study did not infer a clear temporal relationship. However, it is possible that habitual diet and lifestyle of individuals that we observed might not be modified by outcome because colorectal adenoma is asymptomatic. Second, a single measurement of hsCRP may not reflect participants’ long-period status. Also, we cannot rule out the presence of unmeasured or residual confounding factors or measurement error inherent in dietary assessments may exist.

In conclusion, we developed the predicted hsCRP score and found that increasing levels of predicted hsCRP were associated with increasing prevalence of colorectal adenoma in both men and women. Further adjustment for BMI attenuated the association, partly because predicted hsCRP scores was largely explained by adiposity. The associations were more pronounced for advanced adenoma and the magnitudes of associations were modified by age or menopausal status among women. Our study suggests the evidence that diet and lifestyle lowering chronic inflammation may be an important strategy to reduce the burden of colorectal neoplasia.

## Supplementary information

Supplementary table.
