# Supplementary material for: The association between predicted inflammatory status and colorectal adenoma
Source: Sci Rep. 2020 Feb 12;10:2433. doi: 10.1038/s41598-020-59271-1 (PMC7016133; doi:10.1038/s41598-020-59271-1)
Supplement: Supplementary file 1 — Supplementary table. [file 41598_2020_59271_MOESM1_ESM.docx]

**The association between predicted inflammatory status and colorectal adenoma**

Sejin Kim^1^, Sihan Song^1^, Young Sun Kim^2^, Sun Young Yang^2*^, Jung Eun Lee^1, 3*^

Authors’ Affiliations

^1^Department of Food and Nutrition, College of Human Ecology, Seoul National University, Seoul, Republic of Korea

^2^Department of Internal Medicine, Healthcare System Gangnam Center, Seoul National University Hospital, Seoul, Republic of Korea

^3^Research Institute of Human Ecology, Seoul National University, 1 Gwanak-ro, Gwanak-gu, Seoul, Republic of Korea

* Correspondence to Jung Eun Lee and Sun Young Yang

Jung Eun Lee, Department of Food and Nutrition, College of Human Ecology, Seoul National University, 1 Gwanak-ro, Gwanak-gu, Seoul, 08826, Republic of Korea; Tel +82-2-880-6834; E-mail jungelee@snu.ac.kr

Sun Young Yang, Department of Internal Medicine, Healthcare System Gangnam Center, Seoul National University Hospital, 152 Teheran-ro, Gangnam-gu, Seoul, 06236, Republic of Korea; Tel +82-2-2112-5491; E-mail syyang@snuh.org

| Supplementary table 1. Food groups used in the prediction model | |
| --- | --- |
| Foods or food groups | Food items |
| Refined rice | White rice |
| Mixed rice | Rice with black beans, barely, or brown rice |
| Breakfast cereals/mixed grain powder | Breakfast cereals, mixed grain powder |
| Noodles/dumplings | Noodles, instant noodles, noodles in blackbean sauce, spicy seafood noodle soup, cold noodles, dumplings, japchae (korean glass noodles) |
| Rice cake | Rice cake |
| Sandwiches | White bread, toast, sandwiches |
| Sweet bread | Red beam bread, doughnuts, or other sweet breads |
| Pizza/hamburger | Pizza, hamburger |
| Added sugar | Jam, honey, butter, margarine (with bread) |
| Cake/snacks | Cake, cookies, snacks, candy, chocolate |
| Nuts | Peanuts, almonds, pine nuts |
| Soy products | Beans, tofu, soy milk |
| Soup and stew with soybean paste/soybean paste | Soup and stew with soybean paste, soybean paste, seasoning soybean paste |
| Eggs | Eggs |
| Muk | Muk |
| Potatoes | Potatoes |
| Sweet potatoes | Sweet potatoes |
| Fermented vegetable | Fermented vegetables (Kimchi, pickle) |
| Red and Yellow vegetables | Carrots, pumpkin, sweet pumpkin, tomatoes |
| Green leafy vegetables | Chinese cabbage, spinach, lettuce, sesame, cabbage, kale, chicory, bok choy, broccoli, leafy vegetables (shepherd's purse, leaves of beet, mallow, mugwart), bracken, sweet potato stem, taro stem, pepper leaves, short-fruit pimpinella) |
| Other vegetables | Radish, balloon flower, bean sprouts, cucumber, zucchini, onion, green pepper |
| Mushroom | Mushrooms |
| Pork | Pork |
| Processed meats | Ham, sausage |
| Beef | Beef |
| Other meats | Organ, other meats |
| Chicken | Chicken |
| Fish | Fish |
| Processed fish | Canned tuna fish, fish cake |
| Shellfish | Shellfish, oyster, swimming crab, shrimp |
| Fermented seafood | Salted seafood (squid, pollak roe, shrimps, anchovy, shellfish) |
| Seaweeds | Seaweeds |
| Milk/Yogurt/Cheese/Ice cream | Milk, yogurt, cheese, ice cream |
| Carbonated beverages | Carbonated beverages |
| Coffee | Coffee |
| Green tea | Green tea |
| Other beverages | Tea with sugar, sweet drinks |
| Fruits | Tangerine, orange, strawberries, watermelon, apples, pear, bananas, grapes |

| Supplementary table 2. Odds ratio (OR)s and 95% confidence interval (CI)s for colorectal adenoma according to quartiles of actual hsCRP levels | | | | | | | | | |
| --- | --- | --- | --- | --- | --- | --- | --- | --- | --- |
|  | Quartiles of the actual hsCRP levels | | | | | | |  |  |
|  | Quartile 1 |  | Quartile 2 |  | Quartile 3 |  | Quartile 4 |  | *p* for trend |
| Men (n=591) |  |  |  |  |  |  |  |  |  |
| Number of case/noncase | 46/101 |  | 60/90 |  | 66/82 |  | 67/79 |  |  |
| hsCRP, mg/L, mean ± SD | 0.25 ± 0.07 |  | 0.49 ± 0.08 |  | 0.91 ± 0.17 |  | 2.61 ± 1.47 |  |  |
| Age-adjusted model | Reference |  | 1.40 (0.86, 2.28) |  | 1.70 (1.05, 2.76) |  | 1.74 (1.07, 2.83) |  | 0.026 |
| Multivariate adjusted model^a^ | Reference |  | 1.39 (0.85, 2.27) |  | 1.74 (1.06, 2.83) |  | 1.78 (1.09, 2.92) |  | 0.020 |
| Multivariate adjusted model^b^ | Reference |  | 1.25 (0.76, 2.06) |  | 1.45 (0.88, 2.41) |  | 1.42 (0.84, 2.38) |  | 0.193 |
|  |  |  |  |  |  |  |  |  |  |
| Women (n=385) |  |  |  |  |  |  |  |  |  |
| Number of case/noncase | 12/76 |  | 24/93 |  | 23/70 |  | 33/54 |  |  |
| hsCRP, mg/L, mean ± SD | 0.17 ± 0.05 |  | 0.38 ± 0.08 |  | 0.79 ± 0.17 |  | 2.88 ± 1.83 |  |  |
| Age-adjusted model | Reference |  | 1.35 (0.62, 2.92) |  | 1.56 (0.70, 3.46) |  | 2.83 (1.31, 6.14) |  | 0.003 |
| Multivariate adjusted model^c^ | Reference |  | 1.42 (0.65, 3.13) |  | 1.56 (0.69, 3.52) |  | 3.01 (1.37, 6.64) |  | 0.003 |
| Multivariate adjusted model^d^ | Reference |  | 1.36 (0.62, 3.01) |  | 1.41 (0.62, 3.23) |  | 2.59 (1.14, 5.86) |  | 0.014 |
| ^a^ Adjusted for age (continuous, years), alcohol (0, 0-<15, 15-<30, 30≥ g/d), smoking status (past, current, never), regular physical exercise (none, < 14 METs-hours/week, ≥ 14 METs-hours/week), and educational level (high school or below, university or above) | | | | | | | | | |
| ^b^ Adjusted for age (continuous, years), alcohol (0, 0-<15, 15-<30, 30≥ g/d), smoking status (past, current, never), regular physical exercise (none, < 14 METs-hours/week, ≥ 14 METs-hours/week), educational level (high school or below, university or above), and BMI (continuous, kg/m^2^) | | | | | | | | | |
| ^c^ Adjusted for age (continuous, years), alcohol (0, 0-<15, 15 ≥ g/d), smoking status (ever, never), regular physical exercise (none, < 14 METs-hours/week, ≥ 14 METs-hours/week), educational level (high school or below, university or above), and menopausal status (pre-menopausal, post-menopausal) | | | | | | | | | |
| ^d^ Adjusted for age (continuous, years), alcohol (0, 0-<15, 15 ≥ g/d), smoking status (ever, never), regular physical exercise (none, < 14 METs-hours/week, ≥ 14 METs-hours/week), educational level (high school or below, university or above), menopausal status (pre-menopausal, post-menopausal), and BMI (continuous, kg/m^2^) | | | | | | | | | |
